# Performance Optimization of SiO_2f_/SiO_2_ Composites Derived from Polysiloxane Ceramic Precursors

**DOI:** 10.3390/molecules30061385

**Published:** 2025-03-20

**Authors:** Xia Zhang, Bo Xiao, Yongzhao Hou, Guangwu Wen

**Affiliations:** 1 School of Chemistry and Chemical Engineering, Shandong University of Technology, Zibo 255000, China; 18409350572@stumail.sdut.edu.cn (X.Z.);; 2 School of Materials Science and Engineering, Shandong University of Technology, Zibo 255000, China

**Keywords:** polysiloxane, precursor, interface, quartz fiber reinforced ceramic matrix composites, dielectric property

## Abstract

In this paper, polymethylhydrosiloxane (PMHS) and ethanol were used as raw materials to synthesize the ceramic precursor of side ethoxy polysiloxane (PESO) using dehydration and a dealcoholization reaction, which had a ceramic yield of 87.15% and a very low residual carbon content. With the quartz fiber as a reinforcer, the silica matrix composites (SiO_2f_/SiO_2_) with a double-layer interface (PyC-SiO_2_/BNNSs) coating were manufactured using precursor impregnation pyrolysis (PIP). The as-prepared SiO_2f_/SiO_2_ possessed an excellent mechanical property, which exhibited obvious fiber pull-out and debonding phenomena from a fracture morphology. The flexural strength and fracture toughness of SiO_2f_/SiO_2_ reached 63.3 MPa and 2.52 MPa·m^1/2^, respectively. Moreover, the SiO_2f_/SiO_2_ had suitable dielectric properties, with a dielectric constant of about 2.5 and a dielectric loss of less than 0.01. This work provides an important concept for the enhancement of the dielectric properties and mechanical properties of quartz fiber-reinforced ceramic matrix composites, as well as in the preparation of wave-transmissivity composites.

## 1. Introduction

With the rapid development of aerospace electronics, the performance requirements for antenna systems, including radomes on aerospace vehicles, are steadily increasing [1,2,3,4,5]. As the protective shield for the antenna system, the radome must endure the severe aerodynamic conditions encountered during high-speed flight while maintaining a reliable and precise signal transmission [6,7,8]. It serves as a structural component that integrates multiple functions, including load-bearing, wave transmission [9], thermal protection [10,11], high-temperature resistance [12,13], and ablation resistance [14,15]. During high-speed aerodynamic flight, the radome experiences the combined effects of aerodynamic and inertial loads [16]. Consequently, while ensuring excellent wave transmission performance, it is imperative that the radome also possesses adequate mechanical properties [17,18].

In previous research, continuous fiber-reinforced composite materials exhibited a significantly higher fracture toughness compared to single-phase ceramics, enabling them to effectively mitigate sensitivity to cracks and thermal shocks [19,20]. Their advantages, including high specific strength [21], high specific modulus [22], wear resistance [23], and good thermal stability [24], have demonstrated considerable benefits in the aerospace sector [25,26]. Currently, extensive research has been conducted on continuous fiber-reinforced ceramic matrix composites by ceramic engineers. Quartz fiber-reinforced quartz ceramic matrix (SiO_2f_/SiO_2_) composite materials are widely utilized in aerospace vehicle radomes due to their excellent thermal protection and wave transmission properties [27,28]. Following a prolonged development period, these materials have established a comprehensive system comprising three main components: the matrix, the interface phase, and the reinforcement phase (fiber) [29,30,31,32,33].

Amorphous SiO_2_ ceramics, synthesized from organic precursors [34,35], have garnered significant attention owing to their remarkable oxidation resistance [36], high strength [37], and superior dielectric properties [38]. Among the various SiO_2_ ceramic precursors, polysiloxane stands out due to its favorable characteristics, including good fluidity, adjustable viscosity, and a high ceramic yield [39,40,41]. In addition, generally polysiloxanes are known for their low dielectric constants [42]. The organic precursor contains numerous active groups that can undergo secondary reactions during the curing process [43,44], resulting in a highly cross-linked network structure that enhances the ceramic yield [45,46]. In contrast to the preparation of composite materials using silica sol, which necessitates 15 impregnation cycles, the use of organic precursors requires only 5 to 7 impregnation cycles to achieve the complete densification of the composite materials. This approach significantly reduces the preparation time while producing composite materials with high density. Xing et al. [47] prepared SiC_f_/Si–O–C composites by using continuous SiC fiber needle-punched mats as the reinforcement and polysiloxane as the precursor, followed by precursor impregnation and pyrolysis. The phase composition, microstructure, complex dielectric constant, and electromagnetic wave (EMW) absorption properties of the SiC_f_/Si–O–C composites were investigated after annealing at different temperatures. Daniel et al. [48] developed the ceramic matrix for carbon fiber-reinforced ceramic matrix composites using a polysiloxane/boron mixture as the precursor. The standard processing techniques for fiber-reinforced polymer composites were employed, enabling the fabrication of complex geometric shapes.

The interface phase plays a crucial role in the interaction between the fiber and the ceramic matrix, serving as a critical link for the transmission of stress and other information [49,50]. To enhance the strength and toughness of fiber–matrix composites, controlling the fiber–matrix interface (F/C) is of utmost importance. Among the recently proposed interface concepts, boron nitride (h-BN) has emerged as one of the most promising options. The hexagonal boron nitride (h-BN) interface layer possesses a graphite-like structure that not only enhances the interfacial bonding strength between the fiber and the matrix within the composite material but also does not significantly affect the electrical conductivity and dielectric properties of the composite. This makes it a relatively ideal interface phase with substantial application potential and market prospects in the aerospace sector. When bulk BN is exfoliated into few-layer BN nanosheets (BNNSs), its outstanding properties are further improved. Wang et al. [51] prepared h-BN coatings of varying thicknesses on quartz fibers. The results indicated that the h-BN coating alters the direction of crack propagation and effectively reduces the interface shear strength. Du et al. [52] introduced a BN interface phase into quartz fiber-reinforced SiO_2_ composite materials. The experimental results indicated that the fibers experienced significant pull-out, resulting in a distinct ductile fracture, while the bending strength increased from 58.6 MPa to 90 MPa. Specifically, quartz fibers exhibiting low strength and strong interfacial adhesion markedly affect the bending strength of fiber-reinforced composites, although the overall reinforcement effect remains limited. Furthermore, the low-strength unidirectional quartz fibers, combined with a matrix characterized by weak interfacial bonding, result in a constrained reinforcement effect on the tensile strength of the composite material. Previous studies have revealed that the single-layer interface suffers from issues such as inhomogeneity and poor densification, and the organic precursors containing active groups, such as –Si–H bonds, can etch the fibers. Therefore, prior to using organic precursors to fabricate SiO_2f_/SiO_2_ composites, a double-interface coating is employed to establish a suitable interfacial phase on the fiber surface, preventing etching damage to the fibers.

This study employed the precursor impregnation pyrolysis (PIP) process, using sided ethoxy polysiloxane (PESO) as the matrix and quartz fiber as the reinforcement. A double-interface coating is fabricated, consisting of a SiO_2_/BNNSs layer with silica sol as the binder, and fugitive carbon coatings formed via polyimide (PI) thermal imidization on the surface of the quartz fibers. The microstructure, high-temperature mechanical properties (including flexural strength, tensile strength, and fracture toughness), as well as dielectric properties (such as dielectric constant and loss tangent), of the quartz fiber-reinforced composites with double-interface coatings are comprehensively examined.

## 2. Experimental

### 2.1. Raw Materials

Polymethylhydrosiloxane (PMHS, relative molecular weight 344, analytical grade) and silica sol (mass fraction 30%) were purchased from Qingdao Jiyida Silica Gel Reagent Co., Ltd., Qingdao, China. Acidic cation exchange resin (NKC-9) was purchased from Zhengzhou Qinsi Technology Co., Ltd., Zhengzhou, China. Tetramethylammonium hydroxide (TMAH) was supplied by Tianjin Guangfu Fine Chemical Research Institute Co., Ltd., Tianjin, China. B-type quartz needle felt (bulk density 0.67 g/cm^3^) was obtained from Hubei Feilihua Quartz Glass Co., Ltd., Jingzhou, China. The polyimide solution (PI) was supplied by Dongguan Yipin Chemical Co, Ltd., Dongguan, China. Hexagonal boron nitride (h-BN), with an average particle size of 20 μm and a purity of 99.8%, was sourced from Qinhuangdao Yinuo High-tech Materials Development Co., Ltd., Qinhuangdao, China. Sucrose crystals (analytical grade) were obtained from Shanghai Aladdin Biochemical Co., Ltd., Shanghai, China. *N*,*N*-Dimethylacetamide was provided by China National Pharmaceutical Group Co., Ltd., Shanghai, China.

### 2.2. Preparation of Materials

#### 2.2.1. Preparation of PESO

Add PMHS and ethanol into a three-necked flask, ensuring that the molar ratio of the silicone hydrogen functional groups to the ethanol hydroxyl groups is 1:1. Stir the mixture thoroughly. Due to the immiscibility of PMHS and ethanol, the system will appear turbid. At 50 °C, slowly add an ethanol solution of TMAH dropwise to catalyze the reaction. This process will be accompanied by the generation of hydrogen and a noticeable thermal effect. Once gas production ceases, add an acidic cation exchange resin that has been rinsed with ethanol. Introduce the solid resin NKC-9 and stir the mixture to neutralize the alkali catalyst present in the system. After approximately 2 h, filter out the solid particles to obtain a transparent clear liquid, which consists of the product and ethanol. Add the mixture to a rotary evaporator and use it to distill the ethanol under reduced pressure. Collect the distilled ethanol for use in the subsequent reaction. At this stage, the remaining liquid in the rotary evaporator constitutes the synthesized product, thus preparing PESO.

#### 2.2.2. Preparation of BNNSs Using the SAMCE Method

**Figure 1:**
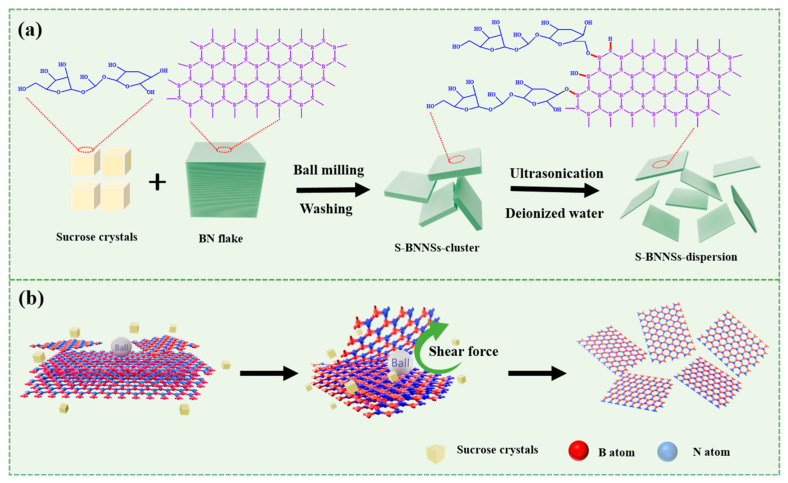
Schematic diagram of the SAMCE process. (**a**) BN exfoliation scheme; (**b**) schematic of ball milling exfoliation of BN.

Functionalized boron nitride nanosheets (BNNSs) were prepared using a typical sugar (sucrose)-assisted mechanochemical exfoliation (SAMCE) method [53], as illustrated in Figure 1. In this process, h-BN powder (3 g), sucrose crystals (10 g), and two ZrO_2_ balls with diameters of 10 mm (100 g) and 1 mm (100 g), respectively, were placed into the ball mill tank. The tank was then rotated at a speed of 150 rpm in a planetary ball mill for solid-state ball milling over a duration of 8 h.

h-BN degrades and thins under the impact and abrasion of small balls and sucrose crystals, reacting in close contact with sucrose powder to produce small, thin BNNSs grafted with sucrose molecules. The ground mixture was washed with 200 mL of deionized water and vacuum filtered through a nylon membrane. This washing and filtering process was repeated four times to ensure the complete removal of free sucrose. BNNSs dispersions were prepared by diluting the slurry in water to create an initial suspension. The mixture was subjected to treatment with a tip sonicator at 40% amplitude for 1 h. Subsequently, the mixture was centrifuged at 2000 rpm for 30 min to remove the thicker BNNSs. The supernatant liquid was collected, evaporated at 80 °C for 4 h, and then vacuum dried at the same temperature for an additional 8 h to obtain the BNNSs.

#### 2.2.3. Preparation of PyC-SiO_2_/BNNSs Double-Interface Coating on the Surface of Quartz Fiber Preform

A specific quantity of silica sol and BNNSs was weighed and mixed uniformly in a ratio of 20:1 using magnetic stirring for 30 min, followed by ultrasonic treatment for an additional 30 min. The resultant impregnation liquid was then fully immersed in a quartz fiber preform in an air atmosphere, subjected to ultrasonic treatment for 30 min, and vacuum impregnated for 1 h. The wet composite was maintained at 80 °C for 2 h to facilitate gel formation. Subsequently, the material underwent blast drying at 120 °C for 2 h to eliminate moisture. Finally, the quartz fiber preform coated with SiO_2_/BNNSs was prepared through sintering at 450 °C for 2 h in an air atmosphere within a furnace, followed by polishing with sandpaper.

Utilize *N*,*N*-dimethylacetamide as the solvent, and incorporate a specific amount of PI to create a solution with a concentration of 5%. Immerse the SiO_2_/BNNSs-coated quartz fiber felt in this solution and subject it to vacuum soaking for 1 h. Following this, the soaked felt is placed in a forced air drying oven. The heating schedule consists of a sequence of temperature increments: 100 °C for 1 h, 150 °C for 1 h, 200 °C for 1.5 h, and finally, 250 °C for 0.5 h, with a heating rate of 10 °C/min. This thermal treatment facilitates the formation of a PI thin film, ultimately resulting in the preparation of a PyC-SiO_2/_BNNSs double-interface-coated quartz fiber preform.

#### 2.2.4. Preparation of SiO_2f_/SiO_2_ Composites

SiO_2f_/SiO_2_ composites were prepared using the polymer impregnation pyrolysis (PIP) process. Quartz-needled felts were used as reinforcement and silica sol was used as precursor of SiO_2_ ceramic matrix. The quartz fiber precursor was first completely submerged in silica sol, sonicated for 0.5 h, and then impregnated for 1 h under vacuum. The wet blanks were held at 80 °C for 2 h to form a gel, blown dry at 120 °C for 2 h to remove the moisture, and sintered in a tube furnace under air atmosphere at 450 °C for 2 h. The above impregnation and sintering processes were repeated to obtain a dense composite material.

#### 2.2.5. Preparation of Quartz Fiber-Reinforced Composites

**Figure 2:**
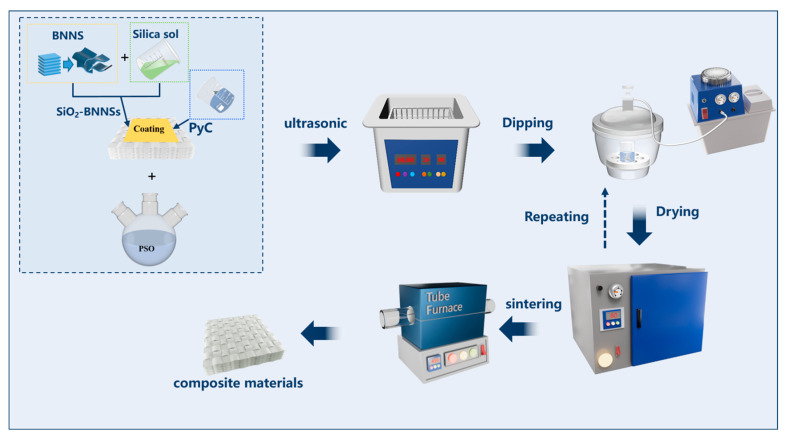
Schematic diagram of SiO_2f_/SiO_2_ (PESO) composites.

The schematic diagram of the preparation of SiO_2f_/SiO_2_ (PESO) composites is shown in Figure 2. The quartz fiber preform, prepared in Section 2.2.3, is immersed in the PESO prepared in Section 2.2.1. Following vacuum immersion for 1 h, the preform is removed and placed in a blast drying oven, where it is subjected to a temperature gradient of 80 °C for 3 h, 120 °C for 2 h, 160 °C for 2 h, and finally, 200 °C for 1 h to complete the curing process. This procedure is repeated until the quality difference of the composite material is less than 1%, indicating that preliminary densification has been achieved. After the curing process is completed, transfer the material to a tube furnace. Raise the temperature to 450 °C at a rate of 2 °C/min in an air atmosphere, maintaining this temperature for 2 h. Subsequently, lower the temperature to room temperature at a rate of 2 °C/min to complete the pre-ceramicization of the PESO. Next, increase the temperature to 600 °C at a rate of 2 °C/min and hold it for an additional 2 h to achieve ceramicization. The preparation of quartz fiber-reinforced silica-based composite materials is then completed, and is followed by analysis and testing.

### 2.3. Characterization

The density of the composite material was measured using the volumetric method. Samples were cut and polished into regular shapes, and their length (*L*), width (*B*), and height (*H*) were measured using a vernier caliper to calculate the volume (*V*). The mass (*m*) of the composite was determined using an analytical balance. This process was conducted on five specimens, and the average result was calculated in accordance with Formula (1).

(1) $$\rho =\frac{m}{V}$$

where ρ (g/cm^3^) is the actual density, *m* (g) is the weight of sample, and V (g/cm^3^) is the volume of sample.

The Archimedes method is widely used to measure the porosity of materials by employing a liquid to infiltrate and displace the open pores, thereby determining the pore volume. In SiO_2f_/SiO_2_ composites, the presence of pores significantly affects the overall volume. Since the Archimedes method cannot directly measure the entire volume, including both the solid phase and all pore structures, it is not suitable for direct density determination. In contrast, volumetric methods offer a more precise measurement of the total volume.

The porosity of SiO_2f_/SiO_2_ composites material was measured using the Archimedean drainage method. Deionized water was used as the impregnating liquid. Before measuring the wet weight of the sample, the sample should be placed into a vacuum pump to maintain negative pressure for at least 30 min to ensure that the bubbles in the sample are completely excluded. The calculation formula of opening rate is shown in Equation (2):

(2) $$P=\frac{m_{3}-m_{1}}{m_{3}-m_{2}}$$

where *P* (%) is the open porosity of the sample, *m*_1_ (g) is the mass of the sample after drying, *m*_2_ (g) is the mass of the sample suspended in water after full immersion, and *m*_3_ (g) is the mass of residual water droplets on the surface of the sample after full immersion in the air.

The mechanical properties of quartz fibers were evaluated using fiber bundle tensile tests, by means of tensile tests with 10 mm specimen length; the loading rate was 0.1 mm/min and each test was performed 10 times.

The evaluation of flexural strength utilized a three-point bending technique implemented on a universal testing apparatus (Instron-1186, Norwood, MA, USA). The samples had dimensions of 3 × 4 × 40 mm^3^, featuring a span of 30 mm and a loading rate set at 0.5 mm/min. The calculation formula of bending strength is as follows:

(3) $$\sigma =\frac{3PL}{2{bh}^{2}}$$

where *σ* is the bending strength of the material, *P* is the maximum applied load during the test, *L* is the span, 30 mm, *b* is the width of the sample (mm), and *h* is the thickness of the sample (mm).

The elastic modulus of the composite can be calculated using the stress–strain curve in the bending strength test according to the slope method. The elastic modulus was calculated as follows:

(4) $$E=\frac{\Delta PL^{3}}{4bh^{3}\Delta s}$$

where *E* (GPa) is the elastic modulus of the composite material, $\frac{\Delta P}{\Delta s}$ is the slope of load-displacement relation curve, *L* (mm) is the span, *b* (mm) is the width of the sample, and *h* (mm) is the thickness of the sample.

The fracture toughness of the composite ceramics was evaluated in accordance with the national standard GB/T23806-2009 [54]. An Instron 5569 electronic universal testing machine was employed for the tests. Polished pre-notched samples measuring 2 mm × 4 mm × 20 mm, with a notch depth of approximately 2 mm, were used. The lower support span was set to 16 mm, and the loading rate was maintained at 0.05 mm/min. Four to six specimens were tested to determine the average value. The formula for calculating fracture toughness is as follows [55]:

(5) $$K_{IC}=\frac{3PL\sqrt{a\times 10^{-3}}}{2BW^{2}}\left[1.93-3.07\left(\frac{a}{W}\right)+14.53{\left(\frac{a}{W}\right)}^{2}-25.07{\left(\frac{a}{W}\right)}^{3}+25.08{\left(\frac{a}{W}\right)}^{4}\right]$$

where *K_IC_* represents the fracture toughness of fused quartz matrix composites (MPa·m^1/2^), *P* denotes the fracture load of the sample (N), *L* is the span length of the sample (mm), *a* signifies the depth of the sample notch (mm), and *B* indicates the width of the sample (mm), *W* represents the height of the sample (mm). All mechanical property measurements were performed at room temperature, with each sample tested eight times to ensure accuracy and obtain an average value.

Under an air atmosphere, a comprehensive thermal analyzer (SDT650, TA, New Castle, DE, USA) was employed to control the heating rate at 10 °C/min, with a temperature range extending from room temperature to 1000 °C. The pyrolysis behavior and thermal effects of PESO were assessed. The structure of PESO at various pyrolysis temperatures was examined using Fourier Transform Infrared Spectroscopy (FT-IR, Model Nicolet 5700, Thermo Fisher Scientific, Waltham, MA, USA) through the KBr pellet method. Nuclear Magnetic Resonance (NMR) is a powerful technique for analyzing material structures. The molecular chain structure of PESO was identified and confirmed through NMR spectroscopy, with deuterated chloroform used as the solvent. The phase composition of the composite was analyzed using an X-ray diffractometer (Rigaku XRD 2500, Akishima, Tokyo, Japan). The dielectric constants of the samples in the X-band (8.2–12.4 GHz) were measured using a vector network analyzer (MS4644A, Anritsu, Atsugi, Japan).

A Hitachi S-4700 scanning electron microscope (Hitachi, Tokyo, Japan) was employed to conduct a comprehensive investigation into several critical aspects of the materials under study. This investigation included an analysis of the morphology, microstructure, and fracture morphology of both the raw materials and the resultant composites. To ensure accurate and reliable observations, the samples underwent a meticulous preparation process. This process involved polishing with sandpaper of varying mesh sizes to achieve a smooth surface, followed by grinding and additional polishing to further refine the samples. After polishing, the samples were thoroughly cleaned with absolute ethanol to eliminate any contaminants. Finally, gold sputtering was applied to the test surface to enhance conductivity and improve the quality of the images captured during the electron microscopy analysis.

## 3. Results and Discussion

### 3.1. Characterization of PESO

#### 3.1.1. Synthesis of PESO

**Figure 3:**
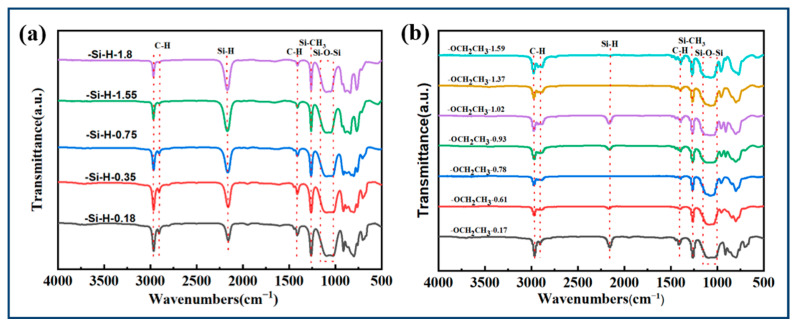
FTIR of PMHS (**a**) and PESO (**b**).

FT-IR spectra was used to determine the functional groups in raw PMHS and the prepared PESO, as shown in Figure 3.

In the FT-IR spectra of the raw material, the characteristic peak corresponding to C–H vibration in the –CH_3_ group is observed at 2967 cm^−1^, while the characteristic peak for Si–CH_3_ vibration appears at 1258 cm^−1^. Additionally, a broad peak in the range of 1000–1100 cm^−1^ represents the vibrational characteristics of Si–O–Si. A distinct vibrational peak is also noted at 2170 cm^−1^, which corresponds to the Si-H group of PMHS. It is observed that as the hydrogen content increases, the intensity of the characteristic peak at 2170 cm^−1^ increases, whereas the C–H vibrational characteristic peak of the methyl group at 2967 cm^−1^ significantly weakens. The infrared spectrum of the product exhibits significant differences compared to that of the raw material. The characteristic peak corresponding to C–H vibration in the –CH_3_ group appears at 2967 cm^−1^, while the peak for Si–CH_3_ vibration is observed at 1258 cm^−1^. Additionally, a broad peak in the range of 1000–1100 cm^−1^ is attributed to Si–O–Si vibrations. Notably, the product lacks a distinct vibration peak near 2170 cm⁻^1^, indicating that the Si–H groups in the system have been nearly completely reacted. Conversely, there is a pronounced vibration peak at 2841 cm^−1^, which corresponds to the C–H vibration characteristic of the –OEt, thereby confirming the successful incorporation of the –OEt group into the PMHS side chain. Furthermore, the intensity of the vibration characteristic peak of the –OEt group in the product synthesized from PMHS is relatively high, while the intensity of the C–H vibration characteristic peak at 2967 cm^−1^ has significantly diminished. The above analysis indicates that the reaction successfully synthesized PEOS from PMHS.

**Figure 4:**
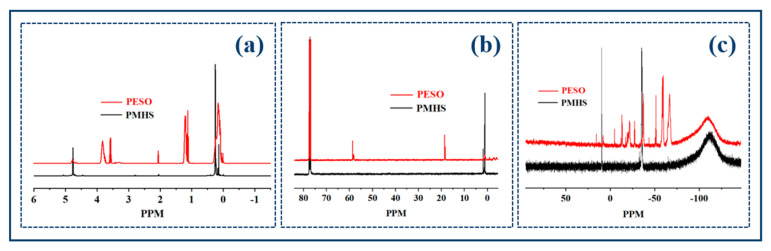
The NMR spectra of PESO. (**a**) ^1^H NMR spectra; (**b**) ^13^C NMR spectra; (**c**) ^29^Si NMR spectra.

To enhance the understanding of the chain structure of PESO, an NMR spectra analysis was conducted, and the results are presented in Figure 4. The ^1^H NMR spectra of the product reveals vibration peaks at approximately 0.05 ppm and 0.15 ppm, which correspond to the characteristic vibrations of H atoms in the –Si–CH_3_ group. Additionally, a chemical shift of about 4.7 ppm is observed, indicative of the vibration of H atoms directly bonded to the Si atom. Notably, the characteristic peak disappeared. The methylene proton peak of the –OEt group was observed at approximately 3.7 ppm. The –CH_2_– proton peak from the –OEt group appears around 3.7 ppm, with the –CH_2_– group of the –OEt coupling with the –CH_3_ group, resulting in a quartet (3.70 ppm) and a triplet (1.24 ppm). This indicates that the –Si–H groups on PMHS reacted with alcohols and disappeared, while –OEt groups were successfully introduced. This observation indicates that the -Si-H group of PMHS reacted with alcohols, leading to its disappearance and the subsequent introduction of the –OEt group. From the ^13^C NMR spectra, PESO exhibits characteristic peaks at −0.97 ppm, −1.07 ppm, 18.66 ppm, and 58.68 ppm, which correspond to the C atoms in the –Si–CH_3_ group, the –CH_2_– group of the –OEt group, and –CH_3_. The ^29^Si NMR spectra reveal signals at δ (ppm) = 15.51, 7.98, −5.15, −13.87, −21.94, −27.61, −37.43, −51.67, −59.81, and −67.02, which correspond to Si-O units in different chemical environments. The peak at δ (ppm) = 15.51 represents the Si atom in the terminal unit of PESO, while the additional peaks to the right indicate the chemical shifts of Si in PESO chains of differing lengths. This indicates that the –OEt group has been successfully integrated with the siloxane chain, resulting in the synthesis of the target product, PESO. However, due to the dehydration and polycondensation processes, which occur under heated air conditions and allow for free reaction, the length of the PESO ‘chain’ is irregular and exhibits a certain distribution. This demonstrates that the reaction effectively grafts –OEt groups onto Si atoms within the PESO backbone.

Based on the combined FTIR spectra and NMR spectra data, the H atoms in PESO primarily exist in the –Si–CH_3_ and the –Si–H. The carbon in the precursor is limited to the –Si–CH_3_, with no other carbon-containing groups. The PESO structure consists of alternating Si and O atoms, forming a large number of Si–O–Si long-chain structures. In conclusion, the synthesized PESO meets the ideal functional group structure of the product, and is a thermosetting resin with Si–O–Si as the repeating structural unit.

**Figure 5:**
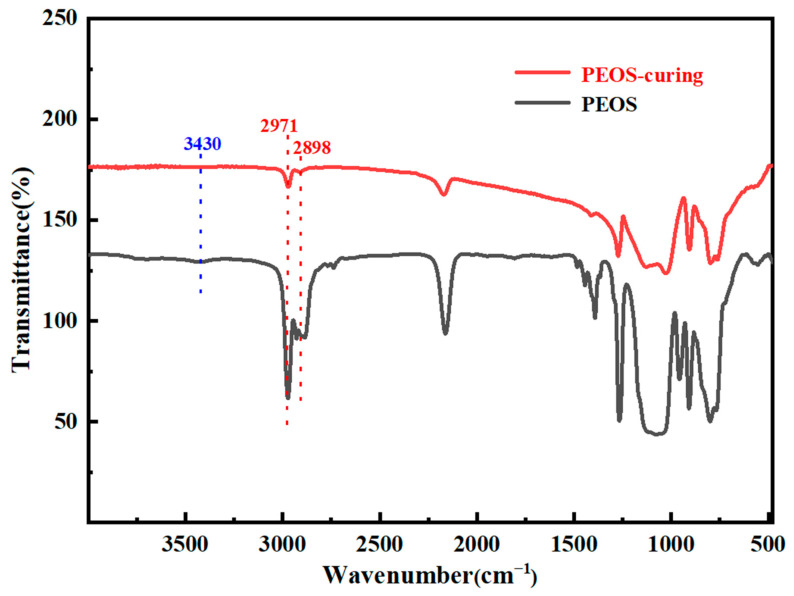
FTIR spectrum of PESO.

**Figure 6:**
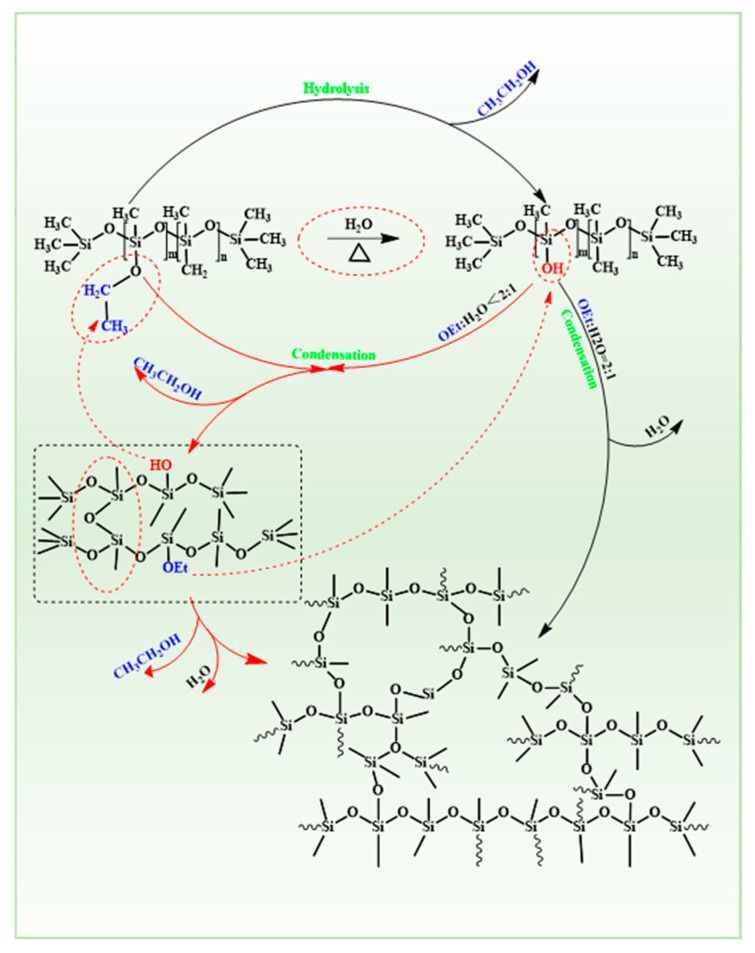
Curing mechanism of PESO.

To further investigate the curing process of PESO, FTIR spectroscopy was utilized to characterize the PESO both before and after curing, as shown in Figure 5. Additionally, the curing mechanism is illustrated in Figure 6.

The FTIR spectra reveal a characteristic C-H vibration peak associated with the –OEt at 2898 cm^−1^. Additionally, a broad peak is observed at 3430 cm^−1^, indicative of the vibration characteristics of the –OH group. Furthermore, the C–H vibration characteristic peak corresponding to the –CH_3_ group is noted near 2971 cm^−1^. Compared with the FTIR spectra of PESO, the intensity of the vibration peak of the cured product at 2898 cm^−1^ is significantly weakened, indicating a reduction in the content of –OEt groups within the system. This suggests that the –OEt groups have undergone hydrolysis and condensation reactions. However, the presence of a weak vibration peak at 2898 cm^−1^ indicates that the –OEt groups of the PESO have experienced a condensation reaction that is not yet complete. Additionally, the –OH peak near 3430 cm^−1^ suggests the presence of residual –OH groups in the system, indicating that the –OH groups formed after the hydrolysis of –OEt groups have not reacted completely. The primary cause of this phenomenon is that, as the cross-linking and curing reactions progress, the degree of cross-linking within the system increases. This increase restricts the movement of the chain segments and reduces the collisions between active groups, resulting in some active groups remaining unreacted in the system’s cross-linking and curing processes.

#### 3.1.2. Curing of PESO

The PESO can undergo a hydrolysis-condensation reaction under certain humidity conditions. This curing reaction requires the participation of water vapor; hence, it is referred to as “moisture curing”. Under heating conditions, water vapor enters the PESO with flowing air as a carrier. The Si–OEt group hydrolyzes to form Si–H groups, with ethanol being eliminated as a small molecule. A dehydration or alcohol elimination condensation reaction then occurs between the Si–H groups, or between the Si–H and Si–OEt groups, resulting in cross-linking and curing to form a hard solid. Due to the high content of –OEt groups in PESO, the solid formed by its cross-linking and curing has a three-dimensional network structure, which exhibits insoluble and infusible properties. Since its main chain is composed of Si–O–Si bonds, it possesses excellent high-temperature resistance. The curing mechanism of PESO is illustrated in Figure 6.

If the molecular weight formed is infinite, it corresponds to SiO_2_. Under these conditions, complete hydrolysis requires 1 mol of –OEt to react with 2 mol of H_2_O, resulting in a ratio of –OH to –OEt of 1:2. When this ratio is less than 1:2, complete hydrolysis cannot occur. The ideal state for cross-linking and curing of PESO is achieved when the –OEt groups are fully hydrolyzed and condensed. However, due to steric hindrance, reactivity, and reverse reactions, it is not feasible for all Si–OH groups to undergo complete condensation. The most common scenario is that the –OEt group is not fully hydrolyzed, and the Si–OH group is not entirely condensed, resulting in a silicone intermediate that contains both –OH and –OEt groups. This intermediate functions as a cross-linking agent. Under the influence of the cross-linking agent, cross-linking occurs between different branches of the chains, leading to the formation of a network structure. As the degree of cross-linking increases and the –OEt and –OH groups are depleted, a hard and brittle transparent solid is produced, thereby completing the curing process.

#### 3.1.3. Pyrolysis of PESO

**Figure 7:**
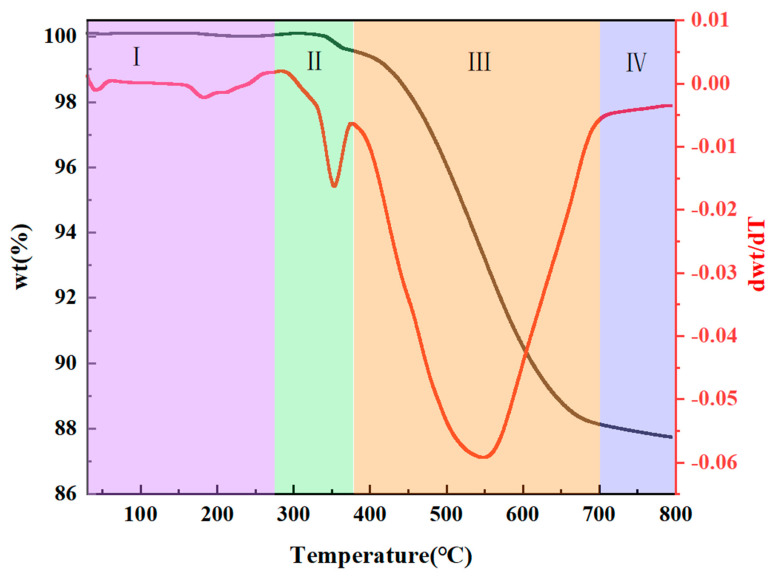
Weight loss curves of PESO in air.

To assess the thermal stability and decomposition behavior of PESO, TG-DTG was performed to track the mass loss of PESO over a temperature range of RT to 800 °C. The results are shown in Figure 7. The overall weight loss process can be divided into four distinct stages.

(1) RT~275 °C: A slight weight loss is observed during this stage, which can be attributed to the release of unreacted small molecules from PESO that has not fully cross-linked during the curing process. Additionally, Si–H groups react with O_2_ in the air.

(6) $$Si-H+O^{•}\to \equiv Si-OH$$

(7) $$\equiv Si-H+H_{2}O\to \equiv Si-OH+H_{2}↑$$

(2) 275~375 °C: During this stage, PESO experiences a gradual weight loss, likely due to further curing and cross-linking, which involves dehydration and alcohol elimination reactions. This leads to the formation of a three-dimensional network structure, reducing the release of small molecules and enhancing the yield of the resulting ceramic.

(8) $$\equiv Si-OH+OH-Si\equiv \to \equiv Si-O-Si\equiv +H_{2}O$$

(9) $$\equiv Si-OH+OH-Si\equiv \to \equiv Si-O-Si\equiv +H_{2}O$$

(10) $$\equiv Si-OC_{2}H_{5}+OH-Si\equiv \to \equiv Si-O-Si\equiv +C_{2}H_{5}OH$$

(11) $$\equiv Si-OH+H-Si\equiv \to \equiv Si-O-Si\equiv +H_{2}↑$$

(12) $$\equiv Si-OC_{2}H_{5}+H-Si\equiv \to \equiv Si-O-Si\equiv +C_{2}H_{6}↑$$

(3) 375~700 °C: In this stage, PESO undergoes rapid weight loss, primarily attributed to the complete decomposition of the polymer network and the removal of residual organic groups. This stage marks the thermal decomposition of PESO, where the material transitions from an organic to an inorganic phase. The Si–O–Si bonds break, and exchange reactions occur between Si–O and Si–H or Si–C, resulting in the generation of gases such as methane.

(13) $$\equiv Si-OH+H_{3}C-Si\equiv \to \equiv Si-O-Si\equiv +C_{2}H_{4}↑$$

(14) $$\equiv Si-OH+H-Si\equiv \to \equiv Si-O-Si\equiv +H_{2}↑$$

(15) $$\equiv Si-H+H_{3}C-Si\equiv \to H_{2}↑+CH_{4}↑$$

(4) 700~800 °C: In this final stage, PESO experiences minimal weight loss as the inorganic conversion is nearly complete. The thermal decomposition process concludes, and the material fully transitions from organic to inorganic, forming SiO ceramic. Ultimately, the ceramic yield of the SiO precursor reaches 87.15%.

- Initiation:

(16) $$\equiv Si-H\to \equiv Si^{•}+H^{•}$$

- Propagation:

(17) $$\equiv Si-CH_{3}+H^{•}\to \equiv Si-{{\mathrm{CH}}_{2}}^{•}+H_{2}$$

(18) $$\equiv Si-{{\mathrm{CH}}_{2}}^{•}+H_{3}C-Si\equiv \to \equiv Si-{\mathrm{CH}}_{2}-Si\equiv {+}^{•}CH_{3}$$

(19) $$\equiv Si-{\mathrm{CH}}_{2}-Si\equiv {+}^{•}CH_{3}\to \equiv Si{-}^{•}\mathrm{CH}-Si\equiv +CH_{4}$$

- Termination:

(20) $$\equiv Si^{•}+\equiv Si{-}^{•}\mathrm{CH}-Si\equiv \to CH{\left(Si\equiv \right)}_{3}$$

**Figure 8:**
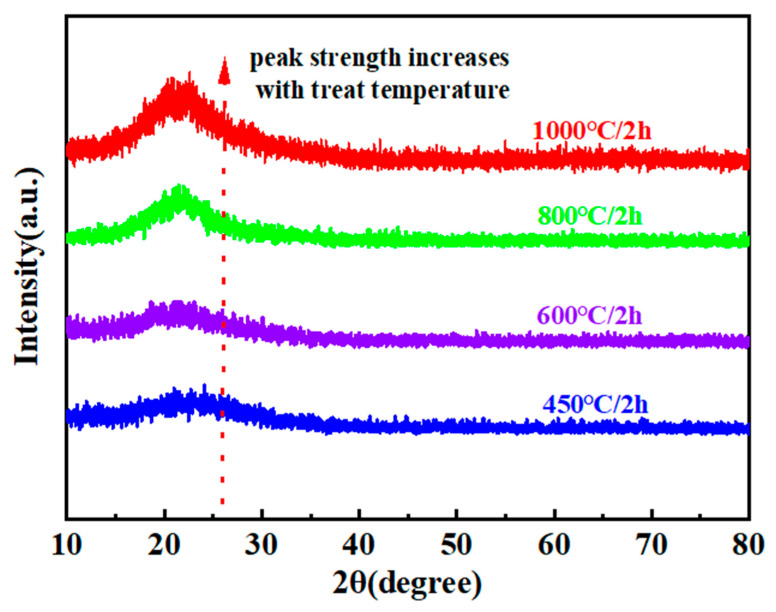
XRD pattern of PESO.

The high-temperature pyrolysis stage plays a pivotal role in determining the properties of quartz fiber-reinforced composites. XRD was used to analyze the structural features of the pyrolysis products of PESO, as shown in Figure 8. In addition, a carbon-sulfur analyzer and a nitrogen-oxygen analyzer were employed to measure the relative elemental composition of the SiO ceramic at various pyrolysis temperatures. The results are summarized in Table 1.

As shown in Figure 8, after treatment at 450 °C, 600 °C, 800 °C, and 1000 °C for 2 h, the material exhibits a broad hump peak near 22°, indicating that the PESO remains in an amorphous state with no distinct crystallization. However, with an increasing treatment temperature, the diffraction peaks show a tendency to sharpen, suggesting that the matrix undergoes crystallization at higher temperatures, which could negatively affect the fiber strength. As the temperature rises, peaks corresponding to SiO_2_ begin to appear. When the temperature exceeds 600 °C, the broad peaks narrow and sharpen, indicating a phase transition and the onset of crystallization. The residual carbon content in the pyrolyzed PESO significantly influences the material’s transmission properties. As shown in Table 1, PESO treated at temperatures of 450 °C and above primarily consists of Si and O, with an exceptionally low residual carbon content. Additionally, the FTIR spectra reveal the disappearance of Si–H bonds and the formation of –OEt groups, indicating that the introduction of –OEt groups facilitates the formation of a cross-linked network through dehydration and alcohol elimination reactions. Based on TG-DTG analysis, the pyrolysis reaction at 600 °C is nearly complete, and the material exhibits overall strength, which is conducive to the formation of high-density composites with excellent mechanical properties, meeting the requirements for subsequent processing. Therefore, considering both the impact of pyrolysis temperature on quartz fiber degradation and the effects of the pyrolysis products of PESO, a pyrolysis temperature of 600 °C is deemed optimal.

**Figure 9:**
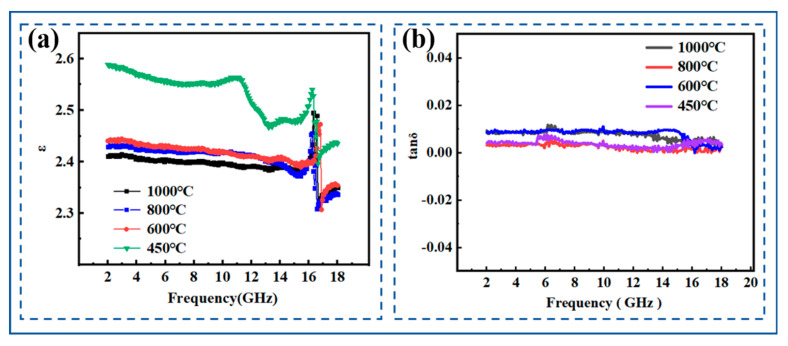
Dielectric properties of PESO. (**a**) Dielectric constant; (**b**) dielectric loss.

Figure 9 shows the dielectric properties of PESO obtained under air atmosphere at different pyrolysis temperatures, within the frequency range of 2–18 GHz. It is evident that both the ε and tg δ remain relatively stable throughout this frequency range, with no significant fluctuations. The gradual decrease in the dielectric constant of the composite material with increasing pyrolysis temperature can be explained from the following perspectives: First, during pyrolysis at 450 °C, the material undergoes structural and compositional evolution. The polymer matrix partially decomposes or transforms into new molecular structures at this temperature. This process generates more polar groups (such as hydroxyl and oxygen-containing groups), which enhance the dipolar polarization capability of the material, particularly in the low-frequency range. Additionally, the molecular chains within the material may break or rearrange during pyrolysis, leading to a more ordered dipole orientation, which further strengthens polarization and results in a higher ε in certain frequency ranges. As the temperature increases further to 600–1000 °C, the organic matrix in the composite material is likely to completely degrade, leading to a significant reduction in the content of polar groups. This reduction weakens the dipolar polarization ability of the material, thereby decreasing the ε. Meanwhile, high-temperature treatment may promote the transformation of some amorphous phases (such as silicon-based materials) into crystalline structures. Compared to amorphous materials, crystalline structures generally exhibit a weaker charge polarization ability, leading to a further decline in the ε.

Although the dielectric constant **ε** of the material undergoes significant changes after pyrolysis at different temperatures, the dielectric loss (tg δ) remains unchanged. This phenomenon can be attributed to the following factors: First, tg δ is typically closely related to the electrical conductivity of the material. If the material’s polarity and structure change during high-temperature pyrolysis, but its electrical conductivity does not significantly increase or decrease, the tg δ may remain relatively stable. Under experimental conditions, the material’s conductivity may not experience drastic fluctuations, resulting in a minimal impact on tg δ. Second, although the ε of the material varies with different pyrolysis temperatures, the fundamental polarization mechanism may remain unchanged. For instance, the dielectric response of the material still primarily relies on the dipolar polarization mechanism. In this case, despite variations in the type and content of polar groups, the overall polarization response remains stable, leading to an unchanged tg δ.

The ε varies between 2.4 and 2.6, and the tangent of the loss angle is approximately 0.01. The material exhibits high electromagnetic wave transmittance and low loss, meeting the requirements for wave transmission performance. The favorable dielectric properties of PESO can be explained using the Debye Equation (21) as follows [56,57]:

(21) $$\frac{\left(k-1\right)}{\left(k+2\right)}=\frac{4\pi }{3}N\left(\alpha_{e}+\alpha_{d}+\frac{u^{2}}{3K_{b}T}\right)$$

where *k* is the dielectric constant, *T* is the temperature, *N* is the number density of the dipole, *α_e_* is the electrode polarization, α*_d_* is the distortion polarization, *u* is the oriented polarization related to the dipole moment, and *K_b_* is the Boltzmann constant.

Low polarity bonds, such as Si–O, generally exhibit low polarization due to the relatively uniform distribution of their electron cloud, which results in a low *α_e_* value. The closely packed cross-linked network further restricts the motion of the polymer chains. The presence of –OEt groups increases the distance between molecular chains. As the temperature increases, the residual Si–OH groups undergo condensation reactions with –OH and –OEt, which further enhances the degree of cross-linking, reduces the –OH content, and limits the motion of the polymer chains. This, in turn, lowers the overall polarization of the polymer, leading to a decrease in both *N* and *α_d_*. The *u* value is typically low in the amorphous structure. The combined reduction in *N*, *α_e_*, *α_d_*, and *u* results in a low *k* value. In the Debye equation, the *u* value (often referring to the Debye temperature) reflects the thermal vibration characteristics of a material. Specifically, compared to crystalline materials, the *u* value of amorphous materials is generally lower. This is primarily due to the absence of long-range atomic order in amorphous materials. The atomic arrangement is randomly distributed, unlike the regular lattice structure seen in crystalline materials. This disorder leads to significant differences in the propagation of phonons (i.e., thermal vibration modes) and their frequency distribution between amorphous and crystalline materials. In crystalline materials, phonon propagation is strongly influenced by lattice vibrations, typically resulting in higher frequencies. In contrast, phonon propagation in amorphous materials is constrained by the random atomic arrangement, which results in a dominance of low-frequency phonons. Since low-frequency phonons carry a lower thermal vibration energy, this leads to a lower Debye temperature and, consequently, a smaller *u* value for amorphous materials. Furthermore, the lack of long-range atomic order in amorphous materials causes a stronger localization of thermal vibrations, meaning that thermal vibrations no longer propagate through the entire lattice in an ordered manner but occur locally instead. This localization effect reduces the heat capacity of amorphous materials, making it lower than that of crystalline materials, which further decreases the *u* value in the Debye equation.

### 3.2. Interface Characterization

Quartz fibers exhibit strong adhesion with PESO resin, forming a robust interface between the two materials. However, after pyrolysis, the mechanical strength of the composite is significantly reduced. Based on our previous research and reference [58], In fiber-reinforced ceramic matrix composites, the interfacial bonding strength plays a crucial role in determining the bending strength. When the interfacial bonding strength is excessively high, the interface rigidity increases, making it difficult for cracks to deflect or slip along the interface. As a result, cracks propagate directly through the reinforcing fibers. Since ceramic materials are inherently brittle, once the fibers are cut by cracks, the fracture mode becomes more brittle, preventing the fibers from effectively toughening the material and reducing the bending strength. Moreover, an overly strong interfacial bond leads to a rapid concentration of stress between the fibers and the matrix, with insufficient buffering effects. This results in a higher susceptibility of the matrix to cracking, accelerated crack propagation, and a weakened overall load-bearing capacity of the composite. Therefore, optimizing the interface design to ensure a moderate bonding strength, which allows for crack deflection and interface sliding, is key to improving bending performance.

In contrast, the incorporation of silica sol and h-BN leads to a notable improvement in the flexural strength of the composite. The formation of a weaker interface facilitates effective load transfer and uniform distribution, resulting in enhanced mechanical strength. Therefore, the addition of silica sol and an appropriate amount of h-BN can improve the load-bearing capacity of the composite. It is worth noting that the flexural strength of the composite described in this study is considerably higher than that reported in previous studies.

An essential step in the preparation of the SiO_2_/BNNSs interface is the fabrication of BNNSs. By exfoliating bulk h-BN into few-layer BNNSs, their excellent properties can be further enhanced. We employed the simple and efficient SAMCE method for exfoliation to prepare functionalized BNNSs. A schematic of the process is shown in Figure 1.

**Figure 10:**
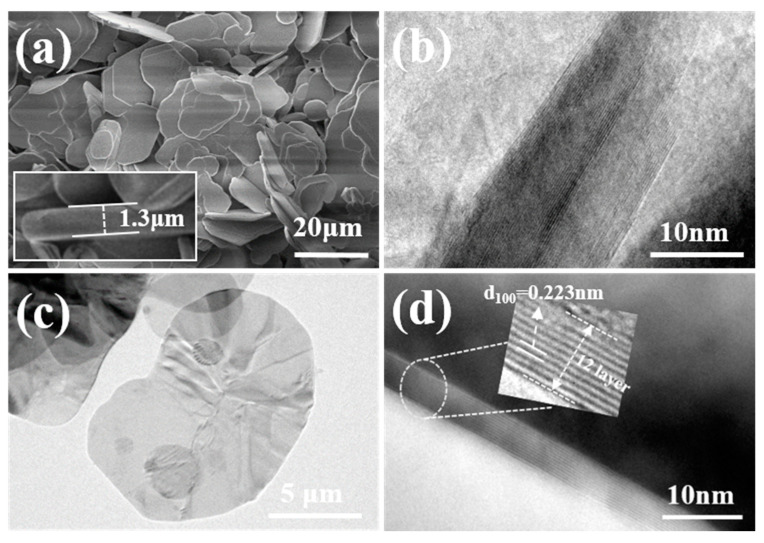
SEM and TEM images of BN before and after delamination. (**a**) BN without dispersion; (**b**) TEM image of h-BN (**c**,**d**) TEM image of BNNSs prepared using SAMCE method.

The morphology and microstructure of BNNSs were investigated using scanning electron microscopy (SEM) and transmission electron microscopy (TEM), as shown in Figure 10. Figure 10a,b show that bulk h-BN (diameter ≈ 20 µm, thickness ≈ 1.3 µm) was exfoliated into thin (<10 nm) BNNSs with approximately 12 layers using ball milling, assisted by sucrose crystals. Additionally, the central region of the BNNSs (Figure 10d) clearly exhibits an ordered lattice structure. The measured distance of 0.223 nm is in close agreement with the (100) plane spacing of h-BN (0.217 nm, JCPDS file number 34-0421) [59], indicating that the crystalline structure in the central region of the BNNSs is largely preserved, facilitated by the sucrose functionalization.

**Figure 11:**
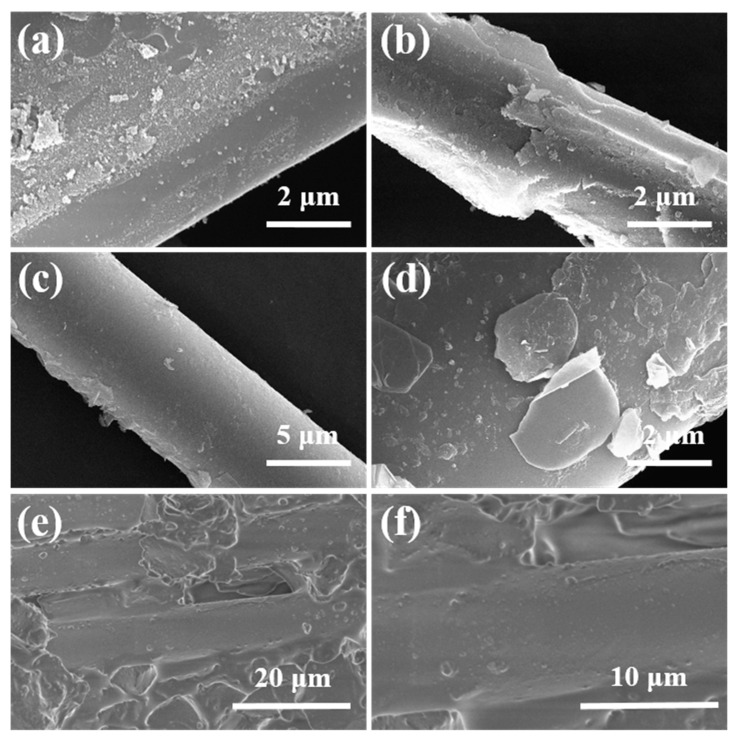
Microscopic surface morphology of coated quartz fibers. (**a**,**b**) SiO_2_ coating; (**c**,**d**) SiO_2_/BNNSs coating; (**e**,**f**) PyC-SiO_2_/BNNSs coating.

The microstructure of the fibers after coating is shown in Figure 11. Figure 11a,b indicate that the silica sol undergoes significant volume shrinkage during the dehydration process, leading to the formation of cracks and preventing the formation of a good interface on the fiber surface. The incorporation of fillers is considered an effective way to reduce the shrinkage of the composites. The use of BNNSs as a filler not only effectively fills the cracks but also weakens the interface, thereby promoting fiber pull-out. Figure 11c,d show a smooth surface without visible cracks, indicating that the interface prepared with BNNSs as fillers provides a complete encapsulation of the fibers. Figure 11e,f illustrate that after thermal imidization, PI forms a thin film that uniformly coats the fiber surface with the SiO_2_/BNNSs layer. The encapsulation of the fibers is intact, which further enhances their protection and reduces the etching effect of active groups in the PESO on the fibers.

Table 2 summarizes the tensile strength and tensile strength retention of quartz fibers with various coatings at elevated temperatures. The results indicate that the tensile strength of the quartz fiber bundles at ambient temperature is 2.77 ± 0.03 GPa. As the temperature increases, a progressive decline in tensile strength is observed. Upon heating to 600 °C, the fibers undergo a significant reduction in tensile strength, with a loss of approximately 50% of the original value. At 900 °C, the tensile strength retention is reduced to approximately 3%, with the fibers exhibiting an almost negligible strength. The fibers with coatings display a similar trend, suggesting that elevated temperatures induce considerable degradation. A comparative analysis revealed that the polyimide coating significantly enhanced the tensile performance of the fibers. This enhancement is attributed to the exceptional film-forming capability and structural integrity of polyimide, which forms a uniform, dense protective layer on the fiber surface, thus providing enhanced mechanical stability.

### 3.3. Composite Material Characterization

#### 3.3.1. Density and Porosity

**Figure 12:**
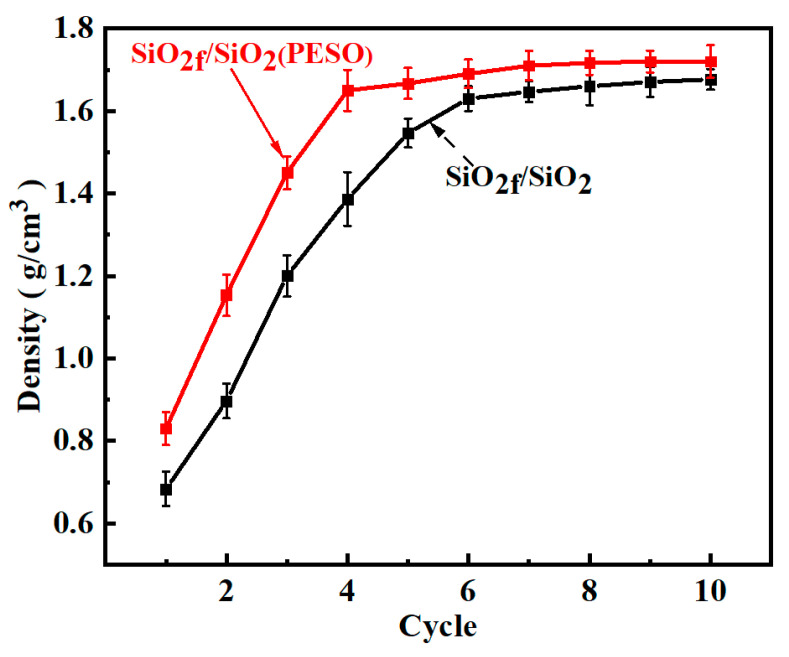
Impregnation time curve for the density of quartz fiber-reinforced composite.

Figure 12 shows the density curve of quartz fiber-reinforced composites. As observed, after seven impregnations with silica sol the density reaches its maximum value of 1.64 g/cm^3^. In contrast, the density of the SiO_2f_/SiO_2_ composites with a PESO matrix reaches 1.65 g/cm^3^ after four impregnations. During the initial impregnation, the liquid precursor rapidly fills the void network, resulting in a high impregnation efficiency. Additionally, the ceramic yield during precursor pyrolysis is relatively high. As a result, the density increase is most significant after the first impregnation. Since the impregnation process is a percolation process, the flow direction of the liquid precursor filling the void network between the fibers is random. Once the percolation threshold is reached, percolation pathways form between the fibers, creating residual voids that are difficult to fill. During the precursor crosslinking and curing process, small molecular gases are released, leaving voids within the product. These voids create space for subsequent impregnation cycles; however, as the number of cycles increases, the pore entrances gradually decrease, eventually forming closed pores that continue to multiply. Consequently, the impregnation solution cannot penetrate these regions, leading to the formation of permanent voids. As a result, the number of accessible voids for precursor filling decreases, causing a gradual reduction in the impregnation efficiency. This explains the slower density increase after the fourth impregnation cycle. Ultimately, the density reaches 1.71 g/cm^3^, significantly reducing the number of impregnation cycles and shortening the preparation time of the composite material.

Combining the data from Table 3 on the density and porosity distribution of the quartz fiber-reinforced composites at different temperatures, the results indicate that as the sintering temperature increases from 400 °C to 600 °C, the porosity of the composites gradually decreases. This is attributed to the increasing shrinkage of the SiO_2_ matrix in the silica sol-based composites, which leads to an enhanced densification and a reduction in the porosity. Concurrently, the interfacial bonding between the fibers and the matrix becomes stronger. During the sintering process of SiO_2f_/SiO_2_ composites, the difference in shrinkage between the fibers and the matrix was a crucial factor. Due to the differing thermal expansion coefficients and distinct shrinkage behaviors of the fiber and matrix materials during sintering, interfacial stresses can develop, which may affect their adhesion. Typically, matrix materials, such as SiO_2_, undergo substantial volume shrinkage at high temperatures, while fibers exhibited a smaller thermal expansion coefficient. As a result, the fibers and matrix experience different degrees of shrinkage and expansion with temperature changes. This disparity in shrinkage can induce significant internal stresses at the interface, thereby impacting the bonding strength between the fibers and the matrix. Excessive shrinkage-induced stresses may lead to interfacial debonding, which compromises the mechanical properties of the composite, particularly the bending strength and toughness. Therefore, when designing composites, it is essential to control the interfacial bonding strength and shrinkage differences. This can be achieved by selecting appropriate fiber and matrix materials, optimizing sintering temperatures, or introducing interface modifiers (such as silica sol and h-BN) to improve the interface compatibility. These strategies help alleviate the interfacial stresses and debonding caused by shrinkage differences, ultimately enhancing the adhesion between the fibers and the matrix and improving the overall performance of the composite. For the quartz fiber-reinforced composites with a PESO matrix, the PESO undergoes nearly complete ceramic transformation at 600 °C, forming a uniformly dense structure, which results in a decrease in the porosity. However, with a further temperature increase, the porosity of both composites begins to rise instead of continuing to decrease. This may be due to the severe shrinkage of the matrix, which generates microcracks.

The shrinkage of the SiO_2_ matrix intensified, potentially leading to the formation of microcracks, which can compromise the interfacial bonding and reduce the bending strength of the composites. Specifically, due to differences in the thermal expansion coefficients of the matrix and fibers, uneven internal stresses develop, particularly at the fiber–matrix interface, where microcracks are likely to form. As the temperature increases, the matrix undergoes significant shrinkage, generating large shrinkage stresses that exacerbate the growth of microcracks at the interface, weakening the matrix’s load-bearing capacity. The mechanical properties of the composite rely on the strength of the interfacial bonding between the fibers and matrix. When the matrix undergoes excessive shrinkage, the interface experiences increased tensile and shear stresses, leading to a reduction in the bonding strength. The initiation and propagation of microcracks cause slight debonding around the fibers, reducing the material’s load transfer capacity. This crack propagation can ultimately result in interfacial debonding, weakening the effective load transfer between the fibers and matrix, thereby reducing the bending strength. The presence of cracks leads to localized stress concentrations, reducing the fracture toughness and making the material more susceptible to failure under bending loads. The expansion of microcracks accelerates brittle failure, especially under high-stress conditions, where cracks propagate rapidly along the interface, leading to a further reduction in the bending strength. Ideally, the matrix should distribute stress uniformly to effectively transfer the load to the fibers, enhancing their reinforcing effect. However, the formation of microcracks disrupted the stress transfer path within the matrix, negatively affecting the overall load-bearing capacity of the composite.

The presence of matrix cracks, even when strong interfacial bonding exists between the quartz fibers and matrix, can significantly compromise the structural integrity of the composite material. Cracks often lead to localized failure, creating regions of stress concentration. Despite good bonding between the fibers and matrix, material failure at the crack site impedes an effective load transfer. As cracks propagate, they cause localized fracture or yielding, which further weakens the composite’s mechanical properties, particularly its bending strength and fracture toughness. In the stress transfer process, the quartz fibers are responsible for efficiently transferring external loads to the matrix. However, crack formation disrupts the matrix structure, causing a discontinuity in stress transfer between the fibers and matrix. Even with strong interfacial bonding, fibers near the crack site cannot effectively transfer the load across the crack, undermining the overall load-bearing capacity of the composite. Cracks can gradually propagate during the material’s use, especially under external loading conditions. This progression not only weakens the matrix’s load-bearing capacity but may also lead to more severe structural failure. Although the fibers may offer some support around the crack, a crack extension can eventually result in the failure of the entire composite, particularly under high-temperature or extreme-loading conditions. Even with strong interfacial bonding between the fibers and matrix, cracks can still induce localized failure at the interface. They cause a stress concentration at the fiber–matrix interface, weakening the bonding strength and accelerating both crack propagation and composite failure. Consequently, matrix cracks significantly affect the structural integrity of the composite, reducing its performance under loading conditions.

#### 3.3.2. Microstructure

**Figure 13:**
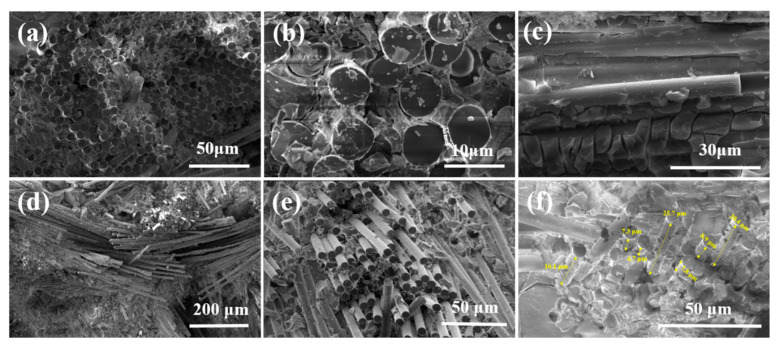
SEM image of the fracture morphology of quartz fiber-reinforced composite. (**a**–**c**) Fracture morphology of SiO_2f/_SiO_2_ composites; (**d**–**f**) fracture morphology of SiO_2f_/SiO_2_(PESO) composites.

Figure 13 shows the fracture morphology of quartz fiber-reinforced composites. As observed in Figure 13a–c, the SiO_2f_/SiO_2_ composites with silica sol as the matrix exhibit smooth fiber ends with minimal fiber pull-out. During sintering, the matrix undergoes significant shrinkage, leading to matrix cracking, while the quartz fibers form a strong interface with the matrix. This results in poor mechanical performance due to the brittle nature of the matrix. In contrast, Figure 13d–f reveal that upon fracture, the fibers are visibly pulled out, with relatively smooth fiber surfaces. This is attributed to the debonding at the PyC-SiO_2_/BNNSs interface, indicating that the interface preparation process was effective and there was no excessive bonding between the fibers and matrix that would cause fiber damage. This ensures that the fiber reinforcement remains intact without being weakened. From Figure 13d,e, it can also be observed that the fibers in the composite are uniformly aligned. When the composite is loaded, fiber–matrix debonding is visible along the fiber axis. This debonding absorbs additional energy, as the presence of fibers causes cracks to deflect under load. The high-strength fibers hinder crack deflection and propagation, thereby improving the overall mechanical performance of the composite. Furthermore, the high fluidity of the PESO matrix facilitates better filling between the fibers, enhancing the density of the composite. During fiber pull-out, the bond between the matrix and fibers resists fiber extraction, and the energy required for fiber pull-out further contributes to the energy dissipation, which in turn enhances the mechanical properties of the composite.

#### 3.3.3. Mechanical Properties

**Figure 14:**
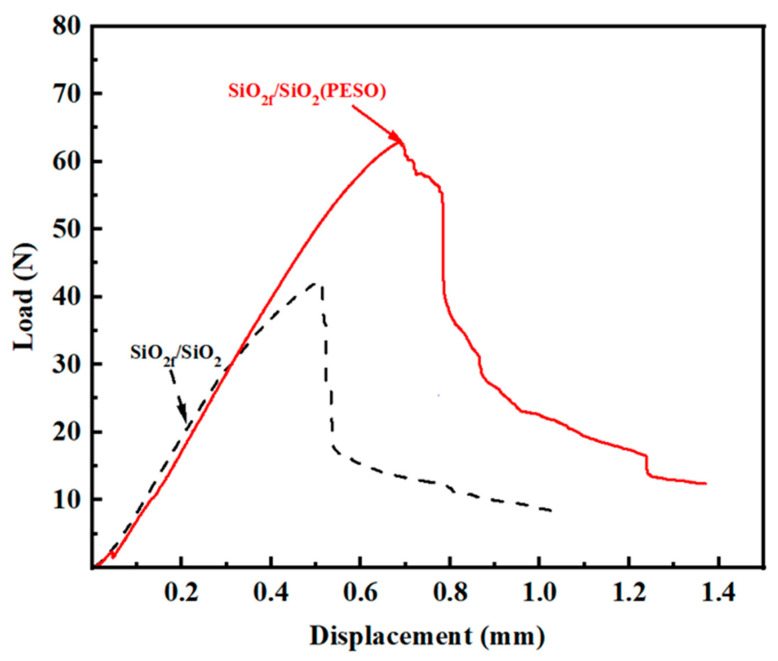
Three-point bending load–displacement curve of fiber-reinforced composites (final processing temperature: 600 °C).

The primary purpose of introducing the coating was to enhance the toughness of the material. Based on the fracture morphology analysis shown in the figure, it can be roughly inferred that the presence of the interface improves the toughness of the composite. To quantitatively assess the change in toughness, both the flexural strength and fracture toughness of the composites were characterized. The fracture toughness was evaluated by calculating the work performed by external forces per unit area from the initiation of loading to fracture. The typical load–displacement curves (after sintering at 600 °C) are shown in Figure 14. By integrating the stress–strain curves, the fracture toughness of the quartz composite at this temperature was found to be 957.3 J/m^2^ for SiO_2f_/SiO_2_ and 3297 J/m^2^ for PESO. As shown in Table 4, the SiO_2f_/SiO_2_ composites prepared using PESO as a precursor for impregnation and pyrolysis exhibited higher flexural strength and fracture toughness after sintering at 600 °C, with values of 63.3 MPa and 2.52 MPa·m^1^/^2^, respectively. Compared to the SiO_2_-based composite, the flexural strength and fracture toughness improved by 54.0% and 6.7%, respectively.

Figure 14 shows the load–displacement curves, which further indicate that the fracture mode is not brittle, suggesting that the fiber–matrix interface strength is moderate. The fibers effectively transfer the load, and fiber debonding and pull-out contribute to the improvement of the composite’s mechanical performance. From the load–displacement curve of the composite, it can be observed that the load and displacement are linearly related when the composite first begins to bear a load. Once the maximum load is reached, the displacement curve shows a distinct stepwise drop with small oscillations (repeated load fluctuations), indicating the presence of the interface. The resistance to crack propagation within the specimen increases, requiring additional energy to be expended. After matrix cracking, the resistance to crack propagation is further enhanced, ensuring the composite retains high mechanical performance and demonstrates typical ductile fracture behavior.

Based on the changes in the composite’s mass gain, density, flexural strength, and fracture toughness, it is evident that the presence of the dual-interface coating significantly enhances the composite’s toughness.

The thermal resistance of SiO_2f_/SiO_2_ composites can reach approximately 1000 °C. However, in experimental studies, 600 °C was commonly chosen as the standard temperature for a performance evaluation due to several key considerations. In the range of 400–600 °C, the material undergoes physical dehydration, the decomposition of functional groups occurs, and there is a structural rearrangement, while it retains favorable mechanical and wave-transmitting properties. At temperatures between 600 and 1000 °C, interfacial stress concentration may arise, weakening the bonding strength between fibers and the matrix and, consequently, diminishing the composite’s flexural strength. Beyond 1000 °C, quartz fibers experience structural transformations, such as partial vaporization or recrystallization, leading to interface degradation. Therefore, 600 °C served as a critical temperature threshold in experimental research, effectively capturing the influence of matrix shrinkage on material performance while preventing a severe deterioration of the fiber–matrix interface, thereby enhancing the reliability and reproducibility of experimental data. In applications such as radomes for hypersonic vehicles, where materials may be subjected to temperatures of 1000 °C or higher, selecting 600 °C as the standard testing temperature ensures stable material performance in a controlled environment, mitigates the risk of unpredictable factors, such as sudden embrittlement or interface delamination during high-temperature testing, and provides a necessary safety margin for engineering applications.

#### 3.3.4. Interface and Strengthening Mechanism of the Quartz Fiber-Reinforced Composite

**Figure 15:**
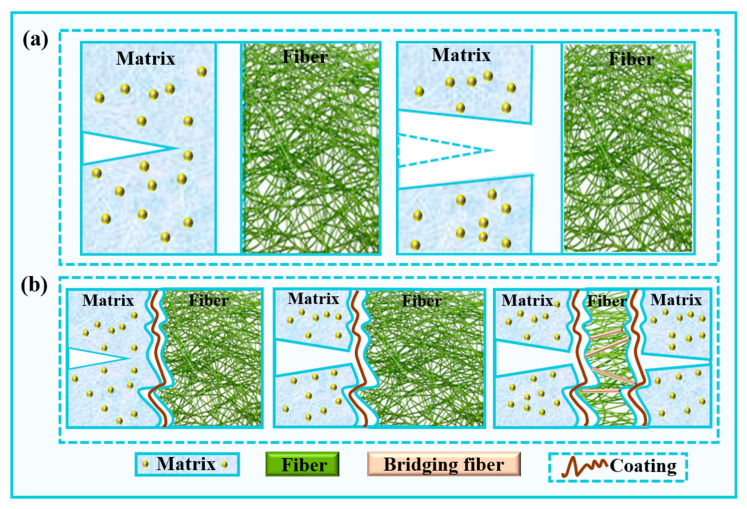
Schematic of load transfer in composite materials with fiber protection coatings. (**a**) Ideal case and (**b**) actual case.

The performance of fiber-reinforced ceramic matrix composites is typically determined using the properties of the fibers, matrix, and their interface, with the interface characteristics having a significant impact on the overall material properties. The interfacial bonding strength controls the energy absorption mechanism, and interfacial debonding, crack deflection, branching, and fiber pull-out all contribute to the improvement of fracture toughness in ceramic matrix composites. When the interfacial bonding strength is too high, it becomes difficult to induce fiber debonding and pull-out, leading to the brittle fracture of the material. On the other hand, when the interfacial bonding strength is too low, the fibers are unable to effectively transfer the load. Only when the interfacial bonding strength is moderate can the fibers effectively transfer load while allowing a certain degree of debonding and pull-out, thereby improving the composite’s strength. A schematic of the load transfer mechanism in the quartz fiber-reinforced composite with fiber protective coatings is shown in Figure 15.

For an ideal smooth gap, as shown in Figure 15a, the fiber remains unconstrained, preventing the load from being transferred from the matrix to the fiber, which results in typical fiber pull-out behavior with a very long length. In contrast, for a realistic gap, load transfer is achieved through a combination of mechanical interlocking, intermittent bonding between the fiber and matrix, and the roughness of both the fiber and matrix surfaces. As shown in Figure 15b, when a crack reaches the interface, the strain in the matrix induces sliding at the interface. Point contact forms between the fiber and matrix, leading to mechanical interlocking and possible bonding. This results in crack deflection, interface debonding, and fiber pullout. The load is then effectively transferred from the matrix to the fiber, enhancing the overall strength of the composite.

#### 3.3.5. Dielectric Properties

Dielectric performance is one of the most important indicators for evaluating the performance of wave-transparent materials, typically characterized by two parameters: the dielectric constant (ε) and the loss tangent (tg δ). In general, quartz-based ceramic materials are considered to have excellent wave transmission properties if their ε is below 3 and their tan δ is lower than 0.01.

**Figure 16:**
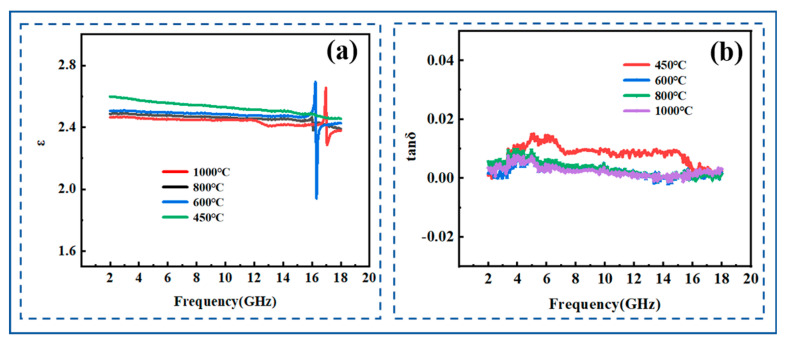
Dielectric properties of SiO_2f_/SiO_2_(PESO) composites after treatment at different temperatures: (**a**) dielectric constant; (**b**) dielectric loss.

Figure 16 shows the ε and tg δ of the SiO_2f_/SiO_2_ (PESO) composites under different temperature conditions. As observed, the composite material exhibits excellent dielectric performance. Within the 2–18 GHz frequency range, both the ε and tg δ remain relatively stable, with the ε centered around 2.6 and the tg δ below 0.01. This indicates that the dielectric properties of the composite material remain stable after various temperature treatments within the 2–18 GHz range, demonstrating outstanding performance that meets the requirements for wave transparency.

The dielectric constant peak observed at 16 GHz in SiO_2f_/SiO_2_ composites primarily arises from the synergistic effects of interfacial polarization, resonance absorption, dipole relaxation polarization, and microstructural characteristics. Under a high-frequency electric field, the difference in dielectric constant and electrical conductivity between the quartz fibers and the SiO_2_ matrix significantly enhances the interfacial polarization effect (Maxwell–Wagner effect). As the frequency of the applied field approaches 16 GHz, the interfacial polarization reaches its peak, resulting in a maximum dielectric constant. Additionally, microstructural features and defects within the composite, such as pores and interfacial cracks, may induce localized resonance effects. When the frequency of the external electromagnetic wave aligns with the resonance frequency, local polarization is notably enhanced, contributing further to the peak dielectric constant. Furthermore, under the influence of a high-frequency electric field in the GHz range, polar groups in the material (such as Si–OH and Si–O–Si) undergo relaxation polarization. Since different polar groups have distinct relaxation times, when the field frequency coincides with the relaxation frequency of a specific polar group, the polarization effect is maximized, leading to a peak in the dielectric constant at 16 GHz.

#### 3.3.6. Thermophysical Properties

Given that the primary application of the SiO_2f_/SiO_2_ (PESO) composites is for medium- to high-temperature components, a low coefficient of thermal expansion (CTE) is essential to reduce the deformation of the radome during operation, maintain shape stability, and ensure a secure connection with the missile body. Additionally, a low CTE minimizes the impact on the power transmission efficiency and aiming accuracy, thereby preserving the wave-transmitting properties of the radome. A low thermal conductivity and a high specific heat capacity enhance the thermal insulation performance, protecting the internal antenna system from high-temperature damage. Therefore, studying the thermal and physical properties of composites at different temperatures is crucial.

**Figure 17:**
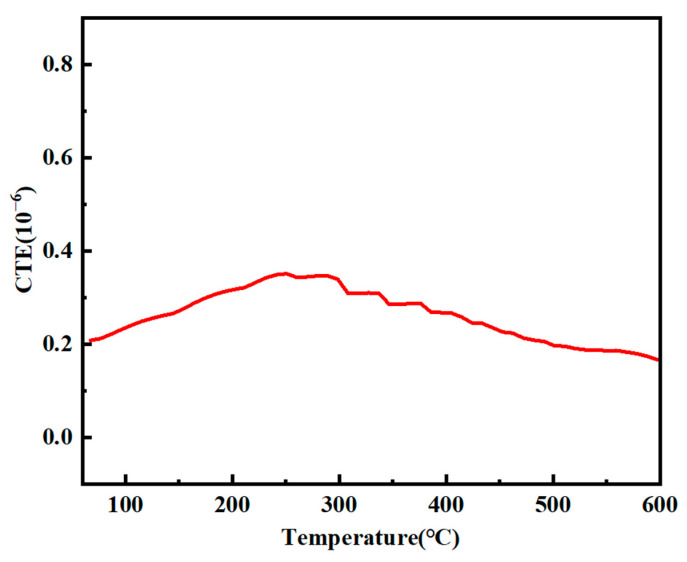
Coefficient of thermal expansion of SiO_2f_/SiO_2_(PESO) composites at different temperatures after various thermal treatments.

As shown in Figure 17, the coefficient of thermal expansion (CTE) of the SiO_2f_/SiO_2_ (PESO) composites increases slightly with temperature. This behavior is primarily attributed to the release of thermal stress between the quartz fibers and the polysiloxane matrix, which causes the CTE to gradually rise as the temperature increases. Since both the quartz fibers and matrix are predominantly composed of SiO_2_, the overall linear thermal expansion coefficient remains relatively low.

The thermal conductivity of the specimens after a 450 °C heat treatment decreases with increasing temperature. As shown in Table 5, after the 450 °C heat treatment, the specimen is in an amorphous state, where its phonon mean free path is approximately constant and significantly lower than that of crystalline materials. Between 500 °C and 600 °C, the PESO undergoes further ceramicization, leading to the formation of more closed pores within the specimen. As a result, the path that heat must travel through the specimen becomes more tortuous, reducing the solid-state heat conduction. Additionally, within this temperature range, the fiber surface treatment agents and coatings begin to decompose and absorb some energy, which further inhibits heat transfer.

In the fabrication conditions described, the SiO_2f_/SiO_2_ (PESO) composites have not reached a crystallized state. Therefore, the SiO_2f_/SiO_2_ (PESO) composites produced under these conditions exhibit low thermal expansion coefficients and reduced thermal conductivity, meeting the requirements for missile radomes.

#### 3.3.7. Moisture Resistance

Due to the presence of approximately 20% porosity in the SiO_2f_/SiO_2_ composite material and the presence of active Si-OH groups on the surface of the matrix, the material is highly susceptible to moisture absorption from the surrounding environment. This moisture uptake can lead to significant changes in the dielectric constant and dielectric loss, thereby impairing the material’s overall performance. Consequently, it is essential to implement a moisture-proof treatment for the composite material to mitigate these effects.

From Table 6, it can be observed that the water absorption rate of the composite material without a moisture-proof treatment is relatively high, reaching over 10%. The sample without moisture-proof treatment is the contrast sample. However, after the coating treatment, the water absorption rate of the substrate is reduced to approximately 0.6%, indicating that the coating provides excellent moisture-proof performance. This can be attributed to the dense arrangement of -CH_3_ groups in the PESO, which transforms the hydrophilic surface of the substrate into a hydrophobic one. The coating forms a pore-sealing layer and a functional moisture-proof layer. The pore-sealing layer effectively closes the surface pores, while the functional moisture-proof layer, with good film-forming properties, ensures an intact coating that prevents the penetration of water molecules, thereby demonstrating excellent moisture resistance.

From Table 7, it can be seen that the coating has no significant effect after being sintered at 200 °C for 2 h. When the temperature is ≤400 °C, the coating consistently maintains good mechanical properties, appearance, and hydrophobicity. However, when the temperature reaches ≥ 600 °C, the coating begins to deteriorate, with most areas undergoing ceramic conversion, and the hydrophobicity of the coating gradually diminishes. When the temperature reaches ≥ 800 °C, the outer waterproof coating is nearly fully ceramicized, and the water absorption rate increases to 8–9%, resulting in almost no moisture-proof effect. This is because, as the temperature increases, the hydrophobic groups in PESO (e.g., –CH_3_ groups) gradually undergo oxidation and decomposition, weakening the moisture-proof performance until it fails.

**Figure 18:**
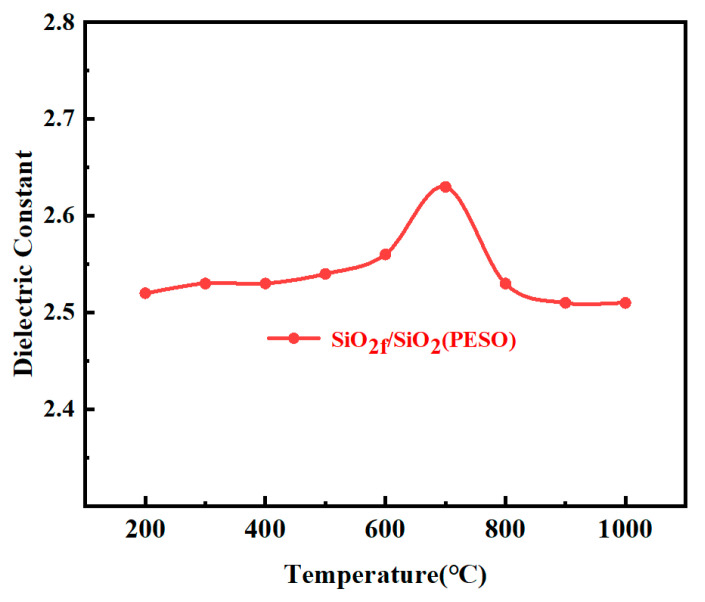
The dielectric constant curve of the SiO_2f_/SiO_2_(PESO) composites surface after the application of moisture-resistant coating.

Figure 18 shows the dielectric constant curve of the SiO_2f_/SiO_2_ (PESO) composites after the preparation of a moisture-proof coating on the surface. The dielectric constant of the coated substrate fluctuates slightly, with little change observed in the dielectric constant of the substrate treated with the coating at temperatures between 200 °C and 400 °C. From 400 °C to 1000 °C, the dielectric constant of the coated substrate first increases and then decreases. Between 400 °C and 800 °C, as the temperature increases, the coating begins to decompose, and organic functional groups are gradually oxidized. The dielectric constant reaches its maximum at 700 °C, primarily due to the insufficient oxygen content during the oxidation process, leading to the presence of some free carbon. At 900 °C to 1000 °C, the coating is fully oxidized, mainly forming silica powder, which has little effect on the dielectric properties of the substrate. In summary, considering the structure of PESO itself, the strong hydrophobic –CH_3_ groups in its molecular structure give it excellent hydrophobicity, effectively preventing the adsorption of water vapor from the air and its infiltration into the material, thereby achieving efficient moisture-proofing.

## 4. Conclusions

As a precursor, PESO significantly shortens the manufacturing cycle of composites due to its adjustable viscosity and excellent flowability, thereby saving both time and material costs. The quartz composite materials fabricated through the PIP process using PESO demonstrate enhanced toughening effects, particularly with the protection of dual interfaces. Experimental results show that the bending strength of SiO_2f_/SiO_2_ (PESO) is 63.3 MPa, and the fracture toughness is 2.52 MPa·m^1/2^, with a fracture energy of 3297 J/m^2^. In comparison to composites with silica sol as the matrix and quartz fibers as the reinforcement, the bending strength and fracture toughness were enhanced by 54.0% and 6.7%, respectively. The composite material exhibited excellent dielectric properties within the 2–18 GHz frequency range, with a dielectric constant varying between 2.4 and 2.6, and a dielectric loss factor of less than 0.01. These properties meet the requirements for wave-transmitting performance. When used in the fabrication of antenna radomes, their outstanding dielectric properties also provide a flexible design space for other process conditions.

## Figures and Tables

**Table 1:** Chemical composition of PESO prepared at different temperatures.

	Si (wt%)	C (wt%)	H (wt%)	O (wt%)
Before cracking	37.1	25.9	6.4	30.6
450 °C	37.23	0.28	1.7	60.79
600 °C	37.17	0.471	1.436	60.923
800 °C	37.35	0.107	0.822	61.721
1000 °C	37.42	0.035	0.536	62.009

**Table 2:** Tensile strength and tensile strength retention rate of quartz fibers with different coatings at different temperatures.

Coating Type	Temperature (°C)	Tensile Strength (GPa)	Tensile Strength Retention (%)
Original quartz fibers	RT	2.77 ± 0.03	
	450	1.74 ± 0.02	63.12
	600	1.31 ± 0.02	47.33
	700	1.07 ± 0.03	38.75
	800	0.55 ± 0.02	20.15
	900	0.09 ± 0.03	3.31
SiO_2_-BN	RT	3.35 ± 0.02	
	450	2.23 ± 0.02	66.56
	600	1.75 ± 0.03	52.23
	700	1.35 ± 0.02	40.29
	800	0.75 ± 0.03	22.38
	900	0.13 ± 0.03	3.83
PI-SiO_2_-BN	RT	3.73 ± 0.03	
	450	2.81 ± 0.02	75.39
	600	2.16 ± 0.02	57.95
	700	1.79 ± 0.03	48
	800	1.17 ± 0.02	31.51
	900	0.14 ± 0.03	3.81

**Table 3:** Density and porosity of fiber-reinforced composites.

Sample	Fabrication Temperature (°C)	Density (g/cm^3^)	Open Porosity (%)
SiO_2f/_SiO_2_	400	1.61 ± 0.03	18.5 ± 0.2
	600	1.63 ± 0.02	17.1 ± 0.3
	800	1.61 ± 0.02	17.5 ± 0.2
	1000	1.59 ± 0.02	17.9 ± 0.3
SiO_2f/_SiO_2_ (PESO)	400	1.68 ± 0.03	17.2 ± 0.2
	600	1.69 ± 0.02	15.1 ± 0.3
	800	1.70 ± 0.02	16.8 ± 0.2
	1000	1.70 ± 0.02	17.7 ± 0.3

**Table 4:** Influences of different sintering temperatures on bending and fracture toughness of fiber-reinforced composites.

Sample	Sintering Temperature (°C)	Bending Strength (MPa)	Fracture Toughness (MPa·m^1/2^)
SiO_2f_/SiO_2_	450	51	2.42
	600	41.1	2.36
	800	11	/
	1000	3.1	/
SiO_2f_/SiO_2_ (PESO)	450	65.7	2.54
	600	63.3	2.52
	800	25.1	/
	1000	3.7	/

**Table 5:** Thermal conductivity of fiber-reinforced composites at different temperatures after heat treatment at 450 °C.

Sample	Treatment Temperature (°C)	Thermal Conductivity (W·m^−1^·K^−1^)
SiO_2f_/SiO_2_ (PESO)	200	0.446
	400	0.317
	600	0.350

**Table 6:** Water absorption of fiber-reinforced composites before and after the application of the moisture-resistant coating.

Sample	Density Before Coating	Density After Coating	Water Absorption	Dielectric Constant
Contrast	1.65	/	10.52	2.38
SiO_2f_/SiO_2_ (PESO)	1.67	1.71	0.4	2.5

**Table 7:** Water absorption of fiber-reinforced composites treated with moisture-proof coating at different temperatures.

Sample	Treatment Temperature (°C)	Water Absorption (%)
	200	0.4
	400	0.61
SiO_2f_/SiO_2_ (PESO)	600	1.21
	800	8.7

## Data Availability

The original contributions presented in this study are included in the article. Further inquiries can be directed to the corresponding author.
